# Common and Unique microRNAs in Multiple Carcinomas Regulate Similar Network of Pathways to Mediate Cancer Progression

**DOI:** 10.1038/s41598-020-59142-9

**Published:** 2020-02-11

**Authors:** Divya Niveditha, Mayank Jasoria, Jayesh Narayan, Syamantak Majumder, Sudeshna Mukherjee, Rajdeep Chowdhury, Shibasish Chowdhury

**Affiliations:** Department of Biological Sciences, Birla Institute of Technology and Science (BITS), Pilani Campus, Pilani, Rajasthan India

**Keywords:** Cancer, Cellular signalling networks

## Abstract

Cancer is a complex disease with a fatal outcome. Early detection of cancer, by monitoring appropriate molecular markers is very important for its therapeutic management. In this regard, the short non-coding RNA molecules, microRNAs (miRNAs) have shown great promise due to their availability in circulating fluids facilitating non-invasive detection of cancer. In this study, an *in silico* comparative analysis was performed to identify specific signature miRNAs dysregulated across multiple carcinomas and simultaneously identify unique miRNAs for each cancer type as well. The miRNA-seq data of cancer patient was obtained from GDC portal and their differential expressions along with the pathways regulated by both common and unique miRNAs were analyzed. Our studies show twelve miRNAs commonly dysregulated across seven different cancer types. Interestingly, four of those miRNAs (hsa-mir-210, hsa-mir-19a, hsa-mir-7 and hsa-mir-3662) are already reported as circulatory miRNAs (circRNAs); while, the miR-183 cluster along with hsa-mir-93 have been found to be incorporated in exosomes signifying the importance of the identified miRNAs for their use as prospective, non-invasive biomarkers. Further, the target mRNAs and pathways regulated by both common and unique miRNAs were analyzed, which interestingly had significant commonality. This suggests that miRNAs that are commonly de-regulated and specifically altered in multiple cancers might regulate similar pathways to promote cancer. Our data is of significance because we not only identify a set of common and unique miRNAs for multiple cancers but also highlight the pathways regulated by them, which might facilitate the development of future non-invasive biomarkers conducive for early detection of cancers.

## Introduction

Cancer has the highest rate in disease-associated deaths worldwide, making it a major public health concern. The global burden of the disease has been increasing ever since, necessitating the need for a better understanding of the disease and facilitating the simultaneous search for appropriate molecular markers for improved prognosis of the disease. The advancements in cancer research have further led to a unified understanding that cancers are heterogeneous in nature; hence, identification and selection of prognostic molecular markers are even more challenging. Parallelly, it is also currently well accepted that the key to successful treatment of cancer would lie in the early diagnosis of the disease. Hence, there is a compelling need for identification of appropriate molecular markers, which could be used as an efficient tool for predicting, diagnosing and monitoring the disease.

Unfortunately, even today, the current gold standard for diagnosis of the majority of cancers is histological examination, obtained by radiologically guided biopsy or surgical excision. However, these procedures are moderately inaccurate, invasive, expensive, and not without the risk to the patient. They are also time-consuming and need to be consistently evaluated by experts. Therefore, there is a clear clinical need for alternative diagnostic techniques. In recent years, there have been a plethora of publications reporting much exquisite molecular information specific to early detection of cancer cells and usage of biomarkers for screening malignancy and recurrence^1–4^. These markers could be amino acids such as Valine, Leucine and Isoleucine as in case of pancreatic cancers^5^, proteins like the CA-125 which are involved in ovarian cancer detection^6^, tumour suppressors like *BRCA1* and *BRCA2* in breast cancers^7^ and so on. In this regard, a steadily growing number of reports have proven that human cancers frequently show an aberrant expression profile of non-coding RNAs, especially the miRNAs.

miRNAs are a class of small non-coding RNAs of 20–24 nucleotide (nt) length, which regulates gene expression at the post-transcriptional level and is conserved across species. These non-coding RNAs interact with specific mRNAs through complementary base pairing primarily within its un-translated region and can inhibit translation of a gene. The evidence indicating the involvement of miRNAs in human cancers started with the discovery of down-regulation of two miRNAs miR-15a and miR-16 in chronic lymphocyte leukaemia^8^ which showed that attenuated expression of these two tumour suppressors leads to cancer. In contrary, up-regulation of certain miRNA clusters like, miR-192, miR-215 and miR-194 showed their involvement in the development of gut tumours^9^. Today, the role of miRNAs as a critical regulator of tumorigenesis has been well established. Hence, in recent years these small RNAs as diagnostic and prognostic markers for cancer are at the vanguard. Another important aspect of miRNAs is their extraordinary stability in the extra-cellular fluid that has positioned them as forerunners as potent non-invasive biomarkers. The vast majority of miRNA expression profiles from solid tumour tissues and body fluids have already indicated that circulating miRNAs originate from tumour tissues which are also protected from endogenous RNase activity^10,11^ reflecting the potential of developing miRNAs as extra-cellular biomarkers for cancer as well as other diseases.

However, despite an ever-increasing significance of miRNAs as future biomarkers for cancer, lack of extensive studies on miRNAs have limited the comparability of data and made it difficult to identify a consistent miRNA signature for the prognosis of cancer. Recently, many computational and prediction methods have been developed to detect miRNAs that are related to cancers^12^ but unfortunately, the performance of these approaches has not been extensively satisfactory. However, concurrently, there has been a rapid pace improvement in miRNA expression profiling technologies and availability of big data in public databases. Till date, these public portals with miRNA expression data from patients across the globe have been absolutely under-utilized, which hence demands further analysis followed by experimentation to confirm the role of miRNAs in cancer.

Hence, in this study, we have analyzed and compared miRNA expression profiles from seven different cancer types obtained from publicly accessible GDC portal. We conducted a differential analysis of miRNA in cancer patient samples compared to tissue-matched control. Importantly, we identified a set of miRNAs that are commonly dysregulated in multiple cancer types. A network of targets and pathways that these miRNAs can collectively regulate was further analyzed to understand the miRNA-mediated regulation of multiple cancers. Next, we asked whether there are any miRNAs that are specific for each cancer type and how their modus operandi differ from that of the common miRNAs. We analyzed the mRNAs regulated by the unique miRNAs specific for each cancer type. Interestingly, this finally led us to the identification of pathways that are regulated by both common and unique miRNAs, which might have implications in carcinogenesis and development of future therapy.

## Results and Discussion

### Identification of common miRNAs among different cancer types

**Figure 1:**
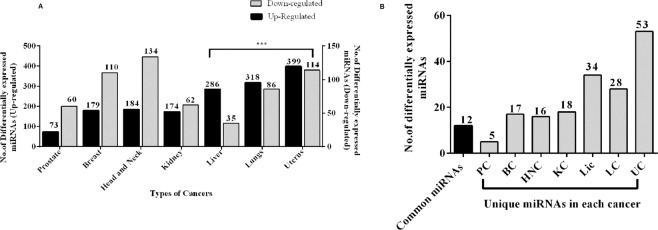
(**A**) Differentially expressed miRNAs in each cancer type. The bar graph represents the up and down-regulated miRNAs. The Y-axis indicates the up and down-regulated miRNAs in each cancer type and the significant ones are indicated in asterisk (*). (**B**) Profiling of miRNAs in each cancer types. The bar graph summarizes the number of common and unique miRNAs in each cancer.

**Figure 2:**
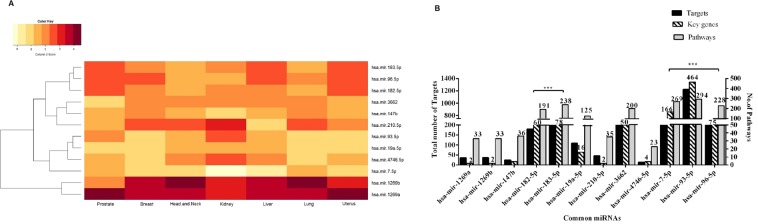
(**A**) The heatmap of common miRNAs in seven different cancer types. The expression profile of the common miRNAs shows up-regulation and darker red indicates higher up-regulation (Fold change < 3). (**B**) A comparative analysis of targets and pathways regulated by common miRNAs. The bar graph represents the number of targets of each common miRNAs, the number of pathways regulated by them and also the number of key genes.

To study the specific role of microRNAs in the seven different cancer types we obtained their miRNA-Seq data from GDC portal. The number of tumour-matched control samples for each cancer type taken under the study is shown in supplementary table 1. For each of these seven cancer types, we wanted to compare the fold changes between tumour and the control samples. Thereby, taking only tumour-matched control samples into consideration will reduce the inter-tissue variations and increases the coherence, which in turn will help in focusing on the changes in the expressions of miRNAs that are involved in cancer progression. Using DESeq. 2 we identified differentially expressed miRNAs between control and tumour samples in each cancer. The number of up-regulated and down-regulated miRNAs obtained is shown in (Fig. 1A). We found that the number of up-regulated miRNAs is high in number in uterus cancer (399), lung (318) and liver cancer (286) when compared with other cancers. A couple of scientific studies have reported that in general miRNAs in tumours were often seen in reduced levels because of genetic loss, epigenetic silencing, transcriptional repression and widespread defects in the signalling pathways^9,13^. This suggests that the presence of many up-regulated miRNAs in these three-cancers (uterus, lungs and liver) of our studies ascertains a dysregulation in expression, which might be involved in the progression of the disease. We next compared the differentially expressed miRNAs across the seven cancer types to identify possible common miRNAs that could be used as prognostic for these cancer types. This approach resulted in the identification of 12 miRNAs, which are common among the seven cancer types as well as unique miRNAs, which are specific to each cancer (Fig. 1B). Interestingly, the heatmap analysis indicated that the expressions of the common miRNAs are univocal (namely differentially up-regulated) across the seven cancers, suggesting an involvement of common regulating mechanism of these miRNAs in tumorigenesis (Fig. 2A). The common miRNAs and their fold change in expression across seven cancer types is shown in supplementary table 3. Furthermore, it was interesting to observe these miRNAs significantly de-regulated with log2 fold change above three in Liver, Lung and Uterus cancer. Thereafter, we assumed that determining the pathways regulated by these miRNAs could aid in understanding the biological significance of the above observation, which in turn will help us in predicting the progression of these cancers.

### The association of common miRNA with corresponding gene-targets and their regulatory networks

**Figure 3:**
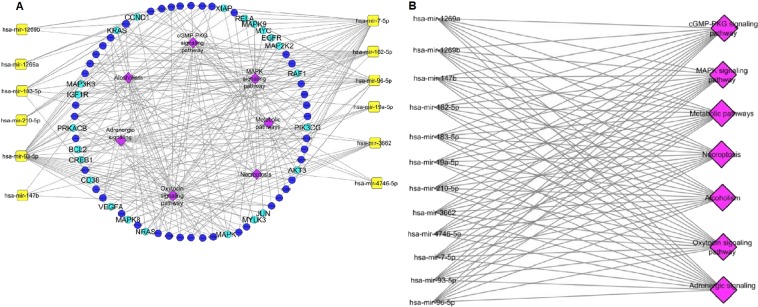
Regulatory network of common miRNAs. **(A)** Cytoscape network showing key genes and pathways regulated by common miRNAs. The cyan colour labelled key genes indicate a dense connection with the miRNAs and the pink colour indicates the pathways being regulated. (**B**) Pathways regulated by common miRNAs.

Since the primary site of each of these seven types of cancer under study is different, the significance of the findings of these common miRNAs would suggest their involvement in alteration of the fundamental signalling pathways in different cancers. To find the regulating mechanism of these common miRNAs, we performed the functional annotation and pathway analysis using KEGG Mapper. A significant number of key genes and pathways were seen to be regulated by five miRNAs (hsa-mir-182-5p, mir-183-5p, mir-7-5p, mir-93-5p and mir-96-5p) whereas miRNAs such as mir-1269a, mir-1269b, mir-147b and mir-4746-5p showed less number of targets (Fig. 2B). The list of key genes targeted by the 12 common miRNAs and their fold change in expression in the seven cancers is shown in supplementary table 4. This data suggests the presence of a positive linear correlation between the number of targets and conservation of miRNAs^14–16^. We assumed that understanding the functional profile of these key genes would be highly informative. Therefore, we constructed a miRNA-gene regulatory network as shown in (Fig. 3A), where each miRNA is linked to the genes it can target. The associated network is merged with gene- pathway network, where a gene is linked to the pathway it is regulating. The resultant merged network gave us a global view of the relation between miRNA-genes and pathways, thereby helping us to understand the pathways regulated by these common microRNAs.

**Figure 4:**
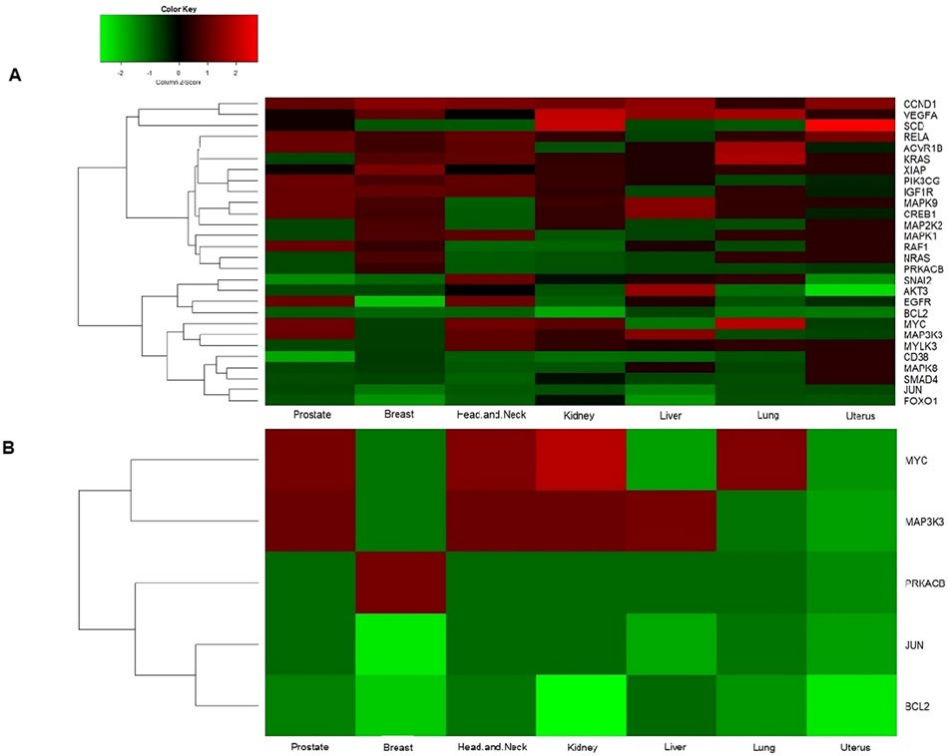
The heatmap of differentially expressed key genes as target of common miRNAs in various cancer types. (**A**) Heatmap of key genes, which are densely connected to common miRNAs. The panel shows down-regulated key genes (green) and up-regulated key genes (red). (**B**) Heat map of common key genes of the seven different cancer types.

We analyzed the interactions of these miRNAs through network analysis and found that they regulate a set of 7 common pathways in the different cancers, which included MAPK signalling pathways, Necroptosis, cGMP-PKG signalling pathways, Oxytocin signalling pathways, metabolic pathways, Alcoholism and Adrenergic signalling (Fig. 3B and Supplementary Fig. 1). The commonalities in the regulation of the seven pathways suggest that the deregulation of these miRNAs was unlikely to be a random event and the genes regulated might be subjected to epigenetic alterations and mutations^17^. For determining that, we analyzed the expression profile of the densely connected key genes (Fig. 4A). We observed that majority of these genes have been reported to be involved in the regulation of myriad cancer signalling pathways, some of which such as KRAS, EGFR and BCL2 have been reported as molecular cancer biomarkers^1,18,19^. While others like, AKT3 and MAP Kinase family genes (*MAP2K2, MAPK1, MAPK8, MAPK9* and *MAP3K3*) which are frequently transcriptionally deregulated kinases, involved in MAPK signalling pathways, p53 signalling pathways and PI3-AKT signalling pathway in most of the cancers^20,21^. Furthermore, we observed that out of all these genes 5 genes e.g. *BCL2, JUN, PRKACB, MAP3K3* and *MYC* were common among the different cancer types (Fig. 4B). The downregulation of *BCL2* and *JUN* suggests a raised feasibility of regression in apoptosis whereas the up-regulation of *MYC* in most of the cancer types shows its involvement in tumorigenesis^22–24^. Further, to understand the involvement of the common miRNAs in the regulation of these genes in each of cancer, we analyzed their regulatory roles.

### The regulatory roles of common miRNAs in cancer

As from the pathway analysis, it was found that the highest number of connections among all the 12 miRNAs was seen in hsa-mir-7-5p, hsa-mir-93-5p and hsa-mir-96-5p. The studies on mir-7-5p have ascribed with multiple roles involving tumour growth, migration, inhibition of the motility and invasion in many cancers making mir-7-5p a promising target for cancer therapies^25–30^ whereas in other studies it was found that mir-93-5p promotes tumour angiogenesis^31^, enhancement of EMT expression^32,33^ and metastasis by suppressing the expression of tumour-suppressor homology2^34,35^. However, the case of mir-96-5p is different as it belongs to the cluster of miR-182/183 which have been reported to be highly conserved^36^ and expressed in a variety of cancers. The pleomorphic functionality of mir-96-5p involves tumorigenesis, cancer progression and metastasis in various cancer indicating its diagnostic and prognostic value in therapies^37^. The members of miR-183-96-182 cluster regulate cell proliferation, apoptosis, metabolism and differentiation by controlling a varied number of signalling pathways^38–40^.

The other common miRNAs such as mir-1269a and b, mir-210-5p, mir-147b, mir-3662-5p, mir-19a-5p and mir-4746-5p suggests that although these miRNAs does not regulate a very large set of genes and pathways, it does have a significant role in the development of cancer as it was seen regulating the most common pathways alongside the highly interactive ones. The up-regulation of the miR-1269a and miR-1269b promotes growth and inhibits apoptosis^41–44^ whereas mir-210-5p over-expression results in an increase of generation of ROS and closely regulates *VEGFR* gene, *Bcl2* which in turn regulates hypoxia and apoptosis in breast and gastric cancer patients^45–48^. A meta-analysis conducted by Li *et al*. and other similar studies showed the prognostic factor of mir-210 in breast cancer, thereby identifying its therapeutic usage^49,50^. The role of mir-3662-5p is still unclear as its expression level is down-regulated in acute myeloid leukaemia whereas its expression increases in lung cancer, and in thyroid cancer shows its regulation of pathways that were related to cancer such as MAPK signalling, TNF signalling, NF-kappa B signalling^51^. This points out that mir-3662-5p can act as both tumorigenic and anti-tumorigenic by reprogramming the metabolic pathways^52^. In the case of mir-19a-5p, it promotes cell proliferation and tumorigenesis^53,54^ whereas a recent study on mir-4746-5p showed its involvement in regulating MAPK signalling, Wnt signalling pathways and TGF-β signalling in lung and colorectal cancer^55,56^.

### The potential common miRNAs as cancer biomarkers

From the above analysis, it was evident that the up-regulation of these common miRNAs was associated with the progression of the carcinogenic process as the regulatory roles of these miRNAs are usually involved in promoting tumorigenesis. In addition, due to their pivot role as regulators of common important signalling pathways in the seven cancer types (Fig. 3B) miRNAs proves to be an ideal therapeutic target. As we did a literature search on these common miRNAs, we understood that most of the miRNAs are reported as potential biomarkers in different types of cancer but none of them has been clinically validated. Therefore, we focused on the properties needed for a miRNA to be a potent non-invasive biomarker. We found out that the foremost requisite is the presence of the miRNA in the system as circulating miRNAs as they can be detected in biological fluids easily, stable outside the cells and are highly specific as they are secreted by the tumour cells^57,58^. Therefore, we submitted our list of common miRNAs to the database mirandola http://mirandola.iit.cnr.it/ to find their classification^59^. We identified that out of 12 common miRNAs four of them such as hsa-mir-210, hsa-mir-19a, hsa-mir-7 and hsa-mir-3662 are reported to be circulating miRNAs^11,60–64^ and other miRNAs such as (182/96/183 cluster along with hsa-mir-93-5p) are reported as miRNAs that are often packed in extracellular vesicles (exosomal miRNAs)^65^.

### Expression profiling of cancer-specific unique miRNAs in different cancer types under the study

**Figure 5:**
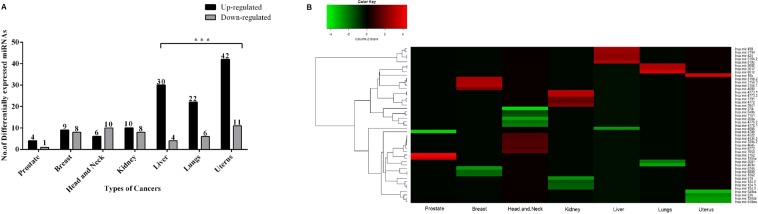
(**A**) Differentially expressed unique miRNAs in each cancer type. The bar graph represents the up and down-regulated unique miRNAs. The significant values are indicated in asterisk (*). (**B**) The heatmap of top 10 differentially expressed unique miRNAs from each cancer type. The heatmap represents the expression profile of unique miRNAs across seven-cancer type where each column is a cancer type. Each row is different miRNA specific to particular cancer. The colour key represents the expression pattern where red is for up-regulation, green for down-regulation and black for no expression.

The above analysis provided insight into the role of common miRNAs in the progression of cancer. Most of the biomarker-based research is focused on the reporting cluster of miRNAs regulating different types of cancers but for better diagnosis miRNAs specific to a certain cancer is equally important. Keeping that in mind, we analyzed the differentially expressed miRNAs for “unique miRNAs” that are specific for discriminating particular cancer. (Fig. 5A) illustrates the number of up-regulated and down-regulated unique miRNAs. In order to study the expression pattern of unique miRNAs, top 10 significantly expressed miRNAs from each cancer type was taken into consideration. The variation in the expression was represented through heatmap (Fig. 5B) which showed that the expression pattern of unique miRNAs was not univocal like the common miRNAs.

### The interaction of unique miRNAs with corresponding targets and their cancer-specific regulatory networks

**Figure 6:**
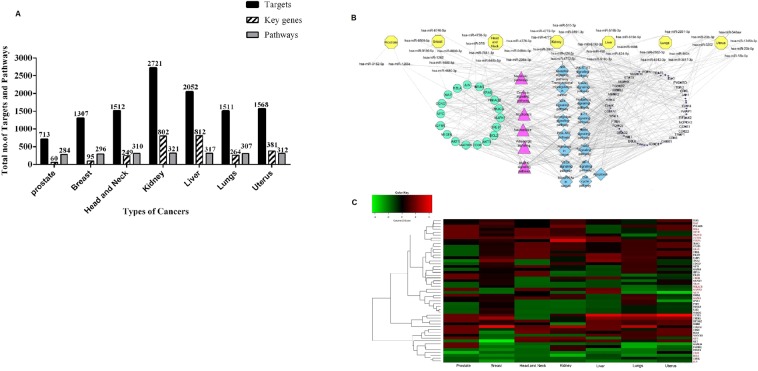
(**A**) A comparative analysis of targets and pathways regulated by unique miRNAs. The bar graph represents the total number of targets and the total number of pathways regulated by unique miRNAs from each cancer types along with the key genes. (**B**) Regulatory network of unique miRNAs. The Cytoscape network illustrates the interaction of each unique miRNAs specific for each cancer type (represented in yellow). The key genes and pathways, which are common for both unique and common miRNAs, are indicated in cyan and pink. The key genes and pathways that specific for unique miRNAs are indicated in blue and black colours. (**C**) Heatmap of differentially expressed key genes of unique miRNAs in the various cancer types. The panel shows down-regulated key genes in green and up-regulated key genes in red. Each row represents the key genes whereas each column represents cancer types. The key genes labelled in red are the common key genes between unique and common miRNAs.

We analyzed the targets regulated by significantly expressed unique miRNA from each cancer type and performed functional annotation based on GO and KEGG Mapper. These led to the categorization of the targets and obtain the key genes and pathways that are shown in (Fig. 6A). The entire regulatory network showing the association between unique miRNAs-genes and the pathways were mapped and analyzed (Fig. 6B). Strikingly, the key genes and pathways regulated by common miRNAs were now found to be regulated by unique miRNAs as well. We compared the common miRNAs to that of unique ones in order to find any correlation in their sequences, family and chromosomal location but we found out that the relationships between these two categories of miRNAs were not as simple as expected. This suggested a presence of a cancer-specific type of relationship thereby, implying the existence of crosstalk within the miRNAs regulatory system.

Furthermore, we found out that the unique miRNAs interact with other key target genes which were mostly identified to be regulating important signalling pathways such as PI3K-AKT signalling, Wnt signalling, JAK-STAT signalling, FOXO signalling and apoptosis pathways. The expression pattern of all the key genes regulated by unique miRNAs (Fig. 6C) suggests that the interactions are highly complex, competitive yet coordinated. Interestingly, we also found that some of the unique miRNAs such as hsa-mir-548b-5p, hsa-mir-208a, hsa-mir-519d and hsa-mir-524 are circulating miRNAs which may serve as biomarkers for specific cancer type^66–68^.

## Conclusion

miRNAs represent an important class of regulators for tumorigenesis as well as a new class of diagnostic and therapeutic target. Till date, in every tumour type analyzed, miRNA profiles of tumour cells are significantly different from normal cells from the same tissue, underlining the biological significance of miRNAs during cancer progression. Moreover, miRNA expression profiles in tumours from similar developmental origins appear to have similar alterations, providing a tool for cancer prognosis and also suggesting that these miRNAs may participate in fundamentally similar signalling pathways altered in many types of malignancies^1,9^. However, despite the fact that an increasing number of studies depict the potential of miRNAs as biomarkers, yet the transition of miRNAs-based biomarkers from bench to bedside has not been smooth. Further, we are yet to specify a signature of miRNAs that are universally dysregulated in multiple cancer types, which might facilitate the transition of miRNA-based diagnostics. Therefore, to improve the reliability of miRNA signatures, it is important to have further stringent studies analyzing sample data in combination with the use of more platforms that are robust, appropriate bio-computational tools and statistical analyses. This will lead to a reduction in inter-study discrepancies and provide us with potent candidate miRNAs as biomarkers or therapeutic targets, which would be highly reproducible and universal.

As we analyzed the miRNA profiles of both common miRNAs and unique miRNAs across the seven cancer types studied, we came across few miRNAs such as hsa-mir-210, hsa-mir-19a, hsa-mir-7 and hsa-mir-3662 which are under the common miRNA category and unique miRNAs such as hsa-mir-548b-5p, hsa-mir-208a, hsa-mir-519d and hsa-mir-524 that are already reported to be present in the circulating fluid, like blood implicating their importance in diagnostics (Supplementary Table 5). Such an observation indicates that the miRNAs identified through our analysis could possibly serve as future prognostic markers for early and easy detection of cancers from blood plasma. However, it is further needed to be validated by future wet-lab studies primarily in clinical samples to confirm the presence of the indicated miRNAs in circulating fluids. This will enable us with an enriched pool of miRNAs that can not only mark the existence of a yet undiagnosed cancer, but also, the unique miRNAs and their presence in circulation can aid the identification of specific cancer types as well. This index system of detection of cancer through miRNAs (both common and specific) might revolutionize future diagnostics and therapy. Importantly, we also observed that majority of the identified miRNAs, both common and unique, were involved in regulating similar pathways like, MAP Kinase, apoptosis, PI3K-AKT which are already implicated in growth and tumorigenesis. These insights might be useful not only in understanding the molecular biology of cancer but might also facilitate the development of future therapies targeting multiple malignancies.

## Materials and Methods

**Figure 7:**
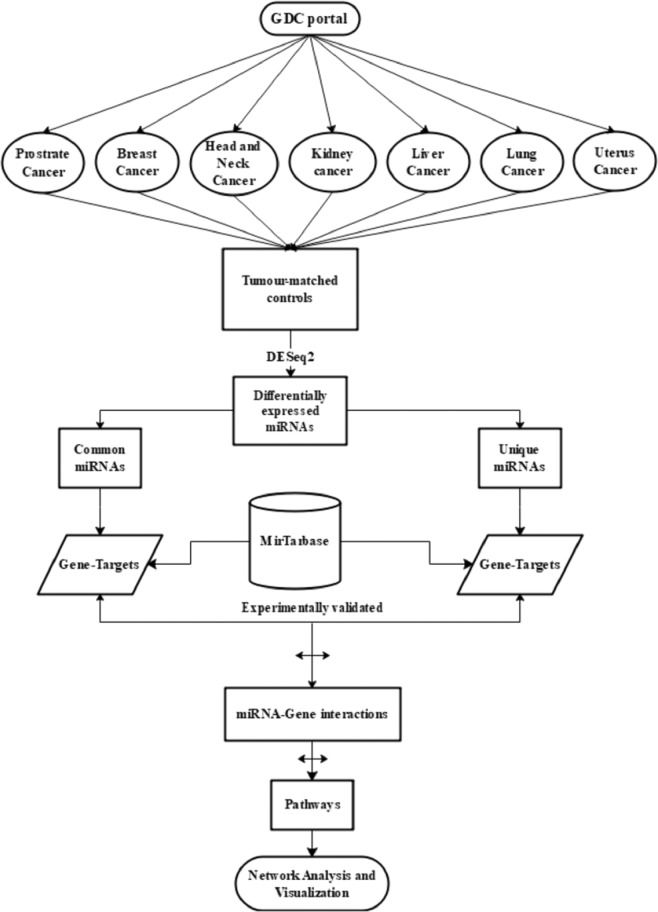
Flow chart. Flow chart depicting steps of data analysis performed.

In this study, we followed two different approaches for studying the miRNA regulatory networks in cancer. Our first approach is to profile differentially expressed miRNAs common to the seven types of cancer under study and find the common pathways they are regulating. The second approach involves obtaining miRNAs, which are specific to each cancer and study their targets and pathways. Finally, the miRNAs of both the approaches were compared to identify the common and specific set of miRNAs-gene network among the different types of cancer under study. A flow chart of this method is shown in (Fig. 7).

### Selection of cancer types for the analysis

For the current study, we selected cancer types belonging to the same developmental origin as members of identical origin often tends to inherit similar properties and expression patterns^1,69^. So, based on cell type and their embryonic lineage (ectodermal and endodermal) the ectodermal/endodermal organ epithelium cancers were subdivided into ten types (squamous carcinoma of skin, lung cancer, colon cancer, breast cancer, pancreatic cancer, head & neck cancer, liver cancer, kidney cancer, prostate cancer and uterus cancer) as obtained from GDC portal https://portal.gdc.cancer.gov/repository. We combined both ectodermal and endodermal tumours as there seemed to be limited difference in their clinical and molecular features in tumours derived from these germ layers^70^. Out of the ten epithelial cancers we finally selected seven cancer types for this study (prostate cancer, breast cancer, head & neck cancer, kidney cancer, liver cancer, lung cancer and uterus cancer) as less number of tumour-matched control samples were available for colon, pancreatic and skin cancers (Supplementary Table 1).

### Data-retrieval using advanced facets/filters to choose genes and miRNAs from GDC portal

The miRNA expression datasets of seven different types of cancers (Prostate cancer, Breast cancer, Head & Neck cancer, Kidney cancer, Liver cancer, Lung cancer and Uterus cancer) were obtained from NCBI Genomic Data Commons (GDC) data portal https://portal.gdc.cancer.gov/repository. We used GDC transfer tool client, a command-line interface which supports a high-performance data handling for downloading GDC data https://gdc.cancer.gov/access-data/gdc-data-transfer-tool. For case filters, we have used the primary sites as our main criteria (prostate, breast, head & neck, liver, kidney, lungs and uterus) followed by TCGA programs; under which projects were PRAD, BRCA, HNSC, LIHC, KIRC, LUAD and UCEC; experimental strategy as RNA-seq; sample type as primary tumour and solid tissue normal; data category as transcriptomic profiling; data type as gene expression quantification and miRNA expression quantification; experimental strategy as RNA-seq/miRNA-Seq and finally the access type was - open-accessed files. The case files having the same UUID for both primary tumours and solid tissue normal were considered and taken as tumour-matched control samples (Supplementary Table 2).

### Data-retrieval from miRNA-seq files of GDC and their expressions in cancers

The miRNA-seq file consisted of 1881 miRNA precursors for each tumour sample and their tissue-matched control samples. The read counts between the tumour-control were compared to quantify the miRNA expressions using DESeq2^71^. The R Package within DESeq2 provided a technique for testing differential expression by using negative binomial generalized linear models, the estimates of dispersion, logarithmic fold changes and incorporating data-driven prior distributions^72^. DEA was conducted separately for each of the seven cancer types using cutoffs of fold change as 1 and p-value < 0.05 for detecting differentially expressed miRNAs in all seven types of cancer.

### Functional annotation of key genes of common and unique miRNAs

After obtaining differentially expressed miRNAs from each cancer type their mature form was identified from miRBase^73^. For each cancer type, we searched for common miRNAs as well as specific ones. For these microRNAs, their gene targets were obtained from miRTarBase^74^. The functional annotation of each target, from the common and unique miRNAs was performed with respect to three aspects based on their Gene Ontology (GO)- intracellular signalling, receptor-mediated signalling and functions regulating diverse other cellular mechanisms. The targets, which fell under all the three functional categories mentioned above, were termed as “Key genes”^75^.

### MicroRNA-Gene network analysis

The Regulatory analysis of miRNAs and their key genes was performed using the Search Pathway tool for the retrieval of interacting genes in each pathway of KEGG Mapper (http://www.genome.jp/kegg/mapper.html) which is a genomic mapping tool and maps genomic content of genes to organism-specific versions of pathways^76–78^. The resultant file comprising the information of pathways being regulated by the miRNA-gene network was converted into nodes and edges and imported to Cytoscape V 3.5.1, an open-source software^79^ for visualizing intricate regulatory networks of the differentially expressed miRNAs and their target genes.

## Supplementary information

Supplementary information
