# Supplementary material for: Common and Unique microRNAs in Multiple Carcinomas Regulate Similar Network of Pathways to Mediate Cancer Progression
Source: Sci Rep. 2020 Feb 11;10:2331. doi: 10.1038/s41598-020-59142-9 (PMC7012856; doi:10.1038/s41598-020-59142-9)
Supplement: Supplementary file 1 — Supplementary information [file 41598_2020_59142_MOESM1_ESM.doc]

**Common and Unique microRNAs in Multiple Carcinomas Regulate Similar Network of Pathways to Mediate Cancer Progression**

Divya Niveditha, Mayank Jasoria, Jayesh Narayan, Syamantak Majumder, Sudeshna Mukherjee, Rajdeep Chowdhury and Shibasish Chowdhury^*^

Department of Biological Sciences, Birla Institute of Technology and Science (BITS), Pilani Campus, Pilani, Rajasthan, India.

***Corresponding author**: [shiba@pilani.bits-pilani.ac.in](mailto:shiba@pilani.bits-pilani.ac.in) (SC),

| **Types of cancer** | **Number of matched Tumour-control samples** |
| --- | --- |
| Breast cancer | 104 |
| *Colon cancer* | 7 |
| Head and Neck cancer | 44 |
| Kidney cancer | 111 |
| Liver cancer | 50 |
| Lungs cancer | 91 |
| *Pancreatic cancer* | 4 |
| Prostate cancer | 52 |
| *Skin cancer* | 3 |
| Uterus cancer | 33 |

**Supplementary table 1. The sample size of the epithelial cancer types with tumour-matched control from GDC portal.**

| **Sl.No** | **Prostate cancer** | **Breast cancer** | **Head & Neck cancer** | **Kidney cancer** | **Liver cancer** | **Lung cancer** | **Uterus cancer** |
| --- | --- | --- | --- | --- | --- | --- | --- |
|  | 00f98c5e-ffce-4338-91ca-da916646797a | 01626fb9-a7f7-4324-97f2-ef2fee03f3c7 | 030988e8-33db-45dc-9a6c-896b55f8c9fe | 000c6bb3-c47d-49a1-ad0f-57093521f922 | 050a903a-7994-4ae1-96c0-e06cf2522b6d | 032f9c8a-572b-427c-bee5-20fed35cf10f | 094b7e36-96de-4b43-824a-80d5eb8732ed |
|  | 012642ed-1618-44a4-9bfc-b607e0178755 | 04248626-f31f-4e3a-aebb-8dca37771ddb | 08bcb7da-fba4-4170-af33-fff85b7a57e9 | 00a05230-dee3-4f71-afaf-640239691532 | 052a2b32-c3cc-4967-b942-1f6272985939 | 03463d0c-fed8-4506-b1b7-d5b9018df525 | 0a03e9ea-fd9b-499a-ba25-7fbb4f076b8e |
|  | 0231472c-49c5-4d8d-9f44-af5e2af66f9d | 053e50eb-7f13-4fed-9264-ee6e9cf86f76 | 0a46d2f1-ca86-48f8-84c4-75b87f5b2a29 | 00be9ee2-1857-4bb4-9d70-1341ef521e1d | 05a7b6b3-e2d6-4320-ad01-94835426aeb6 | 03a93f19-ba03-481e-890a-b4dba8632335 | 0bfb2942-7b15-4907-ab78-a86068607835 |
|  | 03768d6c-d69a-4462-8359-3107688464bc | 069ed9fe-e992-43e8-993f-05bb474ace15 | 0c26388e-4987-493b-95b3-412c11487e77 | 00d551e8-8b6e-49f4-9203-f9834b104edd | 0820d171-84e4-4f2f-91ba-dbbca16bca34 | 05a30195-48d9-4a34-9bbb-8061cc03831b | 0c6734c5-ece0-4ba3-b3ca-a65ae28d6c28 |
|  | 08d249f1-931b-4280-b984-67268ac9737a | 071915bb-9433-473e-91c1-b13bc0ce35d2 | 0e663269-dc69-489e-8a6e-a01cdef55cc6 | 00fa0bb1-bed7-4792-aae6-1cc0687245a4 | 08b92aea-9a12-4110-93a3-f0939eea9908 | 05a8eb54-d450-462c-8c23-c491c70f8d1c | 16408cef-e0a3-4109-b02b-8cdc2a9c0caa |
|  | 0a04717a-1236-4173-b796-3c82e8bd9dc7 | 0759b027-a95e-4679-9262-cd32edbc9490 | 1154dfa7-e96f-4228-b2e5-99c2af7ac63f | 01c1aca9-5756-4f03-b45c-cba47c8c8d75 | 097683f7-5d91-4107-a5b7-1e5ffc2039b7 | 060e230a-e4df-4355-a7bf-e928a68cc4eb | 25c8a6e3-91b1-49c0-90c5-d1609d359f74 |
|  | 0dce275c-4af4-4238-af9e-9100ba200aef | 077413d4-9233-4a64-ac64-ada12cd08dd5 | 14f1dbbe-c23e-45bb-96cb-6ff9b82c90a2 | 02036944-7cc2-422e-9659-b3a208cf036a | 0b66effa-9077-416e-9563-1fad332f9a2d | 069b9873-fac5-425a-bc75-6e362eccef7e | 2f6100df-0399-4026-83c5-02e36bfffc69 |
|  | 0f505546-10a6-4ee8-a73a-3c499b2e75d3 | 07ff7aa3-a3be-44c0-89d8-c67a0dedcc72 | 159e4aac-ed2d-4afa-965a-489fa604160a | 023ab2de-ae1b-4335-a8ca-39b2677a3046 | 0cb6c6e1-d1ab-4e2e-93ea-80e2ea663a50 | 077c2ca3-a580-4798-b361-e7ea2b207b43 | 3a95f223-8916-4468-8a88-af3b0aa15ce6 |
|  | 0f9d7cc6-b241-4fc9-a573-f78a0870e46f | 09ace4e7-5c45-4492-b97c-46aa467820aa | 1731524f-51a2-4631-a6ed-539b5cc77c37 | 02871cb4-f97c-4d34-be0d-97b0ced290bd | 0d2141c1-c26d-4ba3-9728-9715bd715cf0 | 080b040b-3e09-4161-ac0d-b124bf94e87f | 3d7106a8-6fb5-41f0-a5e7-06b32a23c28b |
|  | 0fcce832-d74b-44fb-8535-9438348430a2 | 0a29f991-7e48-4d51-9cd1-f3b26e627ff5 | 17ba443b-66de-4c8a-9725-2d4dce694888 | 050874c3-8eb4-4f88-ba18-58294bd7ede1 | 0e49336b-5f81-433d-b161-d0519832a0b7 | 09106db2-4b23-494a-9418-cd1afeddea62 | 425fe507-343f-40a1-ab60-c5978a282c08 |
|  | 122ee50b-2adb-47bd-b2f1-7668828d501c | 0b64742b-b99d-4fc1-bfbf-bb7074f21a67 | 18e6cee3-4d85-4f4c-8ecd-af7692e74ac0 | 05519481-9138-492d-a544-12651c517ac8 | 101f1660-314b-433e-825f-2931991d6824 | 0920f202-b741-49f8-8a5f-232d41e611f1 | 44f47155-3ad0-42df-832b-67c5112658c2 |
|  | 1404c328-e4cd-4e98-b79b-6c8fcd2a26b8 | 0b9c4345-bf3a-4de7-a022-4039f93d7b27 | 192d9b2f-5591-43d7-b150-83cc1e501f60 | 0604e2f9-0054-41c2-8594-a59ebec039bd | 10c13305-fa56-429d-880a-2827f64503ae | 0a59e31d-5e82-4078-959c-cfe6d773d4c4 | 45758be3-0168-4117-a979-74cb1d8ea838 |
|  | 148fac80-e14e-4f0c-82ee-4d973f732bc4 | 0c6dd343-cf69-4a93-a304-3afc9b287a5e | 1b91276d-fcf4-4c21-b553-dec97a177a1f | 06ad2496-b519-4c4a-a4b6-a86bc6d3f701 | 118ec61b-2bab-40cb-9b57-2ae8ad52f192 | 0a5c12f9-09d3-47fb-b80e-14a09c5bbcf6 | 4a652490-a734-4067-8b64-4d527f1b2d79 |
|  | 149dc1db-9af8-4113-a5a5-e06272468bf4 | 0cd9169f-e533-4da9-8ed4-e56435fa4dc7 | 1cf1f353-bbd4-4acb-9170-87e14fc7f930 | 0752c505-690a-46c8-897d-57566e559182 | 1456ab29-8dd8-41c4-a09b-46de3a02ca15 | 0b94136c-d1f2-4a92-90ad-18c02eec2736 | 4be3db8c-df76-4884-925b-b6b94985408e |
|  | 14c0c865-002d-499f-b8a7-19c4840104c8 | 0d11e213-5ddf-4d0a-9987-f9d0a3056f3a | 1f1bf914-5268-4842-a978-de520d0c9dbf | 0781c269-0f36-4334-ba9d-bc6df0952a7e | 155ad7f3-f30d-4c0a-abea-46cc993e280f | 0c52194f-4f82-4a78-94d8-e109a5d69fc4 | 5426d683-af0a-4123-a218-eeda6c00a478 |
|  | 16cacfe9-4f47-4fec-b29a-af3bcfecad87 | 0db85fe0-94d6-4b8d-bff4-d69aea8a0806 | 20396d1b-eeea-43d2-8b5a-8943bf6846e5 | 079b73c4-e100-4966-bcc7-d87e050485e8 | 18a69d06-66e2-4592-a330-61874a61a887 | 0d3d41b0-a9fa-43cb-baa5-969034619430 | 5beba71e-10a5-49ad-b3dc-113ca46b7f53 |
|  | 1c072746-3cf8-40da-9fc1-9786aa0ff7fe | 0dfa1f7b-98f0-459e-b3bb-29ac447bee53 | 230c7b6b-c382-40f0-bc05-51619678fe62 | 07a526de-cf8d-41b8-a294-23307003f784 | 19aca169-082b-4d27-8d75-55e47d3ce069 | 0d8a058e-bc98-432f-a488-a73eb29de066 | 5c131cb7-5c09-4cc2-84fc-20f279bbcc5c |
|  | 1d15ef76-b8b2-4610-913b-48a5deead997 | 0f9833f9-3923-46cc-ac42-506c5e9e635f | 235b44e7-a567-41c9-84de-cc90be6115f5 | 07ddf5a4-6b38-4622-bdfc-f8bc810c7a38 | 1b4fb31d-6fb7-4ea4-8f89-d43dfce91c29 | 0e109327-0a5c-47ce-8bc0-ccbde85cf8ec | 5f259d38-835e-4d88-a3ed-930640afee45 |
|  | 1d4959d5-7409-49d1-af7e-dbc5c507113a | 10273809-ad79-4848-a896-03f0cefe6861 | 27854e22-3f99-4203-81b0-d2b89471d0cb | 08cfe48a-02c1-4a13-9a07-c816ab118039 | 1d769e29-4188-4e32-a5a1-6ea9f04fb97c | 0f61b059-b204-4190-b4df-d95c31c772a6 | 61b768f5-249d-42dd-9af3-da024a71d250 |
|  | 00f98c5e-ffce-4338-91ca-da916646797a | 10dcd71c-7ed9-43b5-a227-e1249f595b10 | 2aeca07e-28c2-4c71-8d1d-4b22d1b0ecf1 | 09f848a8-4050-406f-bd09-f44ce96f4add | 1e4fe2f8-f758-4653-86f9-5f35e6b694a8 | 0fc4aa84-e6fe-4e4d-b68d-06f09f0201a2 | 6309bbbb-d522-424a-b2e1-c006a0aa8fbd |
|  | 22425080-c93f-4e9a-baf7-b694ed90d1db | 119c02f2-2951-492e-bbe1-1e7120ec4a84 | 2b26a7af-5c33-4206-82f7-99255b2f3936 | 0b0fea8f-6401-4c91-86fd-3dea76a898e9 | 1e78c8a5-aed7-4cf7-8c99-ba2220053948 | 109e561d-d4e9-4b10-8a19-66d4662ccb28 | 67110466-ef17-4c5a-86d6-ad71588617a2 |
|  | 231bb1ae-f7b7-4af0-9eee-61221404885b | 133ae78e-fd83-4a13-9de6-28dbbe8e37c7 | 2c5eacad-0a4f-43e6-ad8f-dae2ffe4b0c6 | 0c1df77d-ecb9-45b7-b680-981d68ef9c09 | 1e810a71-0d10-4a88-a98a-34d22bb2e8fa | 109f4bb5-a959-4171-8822-8115b9282afb | 6830c1ec-b063-4555-b6fb-ffdf3606d4e9 |
|  | 23ae580e-bf2e-4dfc-9d51-b204bed58265 | 144c5bf9-5214-4911-a403-01f9b479ab63 | 2d745b1b-c3cd-41da-ada2-e43c37c83b78 | 0c5ebdcf-16c1-49aa-80f7-ff4a5d0230b0 | 1e9ee1ce-e05a-4dc2-9718-c3a9d24a2916 | 11310889-fb20-481c-957e-1d76486ee453 | 6848ccad-dbc3-4d98-a758-8951e1167bbe |
|  | 245166d8-cb1b-4c4e-b40b-76bfe51565c8 | 14b87f56-49b4-4c5a-8f98-6f02e74601e4 | 2d7c093d-98a4-4b0b-9371-5127098c35be | 0cc9d8bd-ede7-453d-80ad-36ea36051b5c | 1f30abe3-dd5e-4112-921a-e472dc4ded34 | 1625d700-8c20-400d-b337-be8459e8a7cf | 694452fe-3fd1-4bc9-b981-8929e88bbb04 |
|  | 26bb5c96-82bd-403f-b8db-b9a64eba6efd | 153f3345-4527-454e-9aa4-1cdde6d90512 | 30fb36cf-e91f-40b7-94cd-d4cb2aa2c6ae | 0cf93132-5ee6-4b6b-9d47-480c44ba0664 | 20ca035a-6bfa-4daa-bec8-5cdfc1449025 | 16e1e65c-37bd-4ee2-b51f-6c30ae8f30bf | 6f8af8dc-d25b-4212-8934-c40032e3dc70 |
|  | 29ff35b1-6652-412a-b972-5a4f03da41e4 | 1540ed84-19b0-49d5-a728-3b04a29abe8a | 32939f19-c816-45ce-b32a-83aa2102c2f5 | 0d4f93cf-1cec-4315-a61f-14d355479378 | 236d797d-a4c7-4790-a690-bdc96e301b3f | 184d4fe7-0939-42f1-8a06-c78fe6c35ca0 | 75cb033e-c73d-4b5e-a7d3-85de0bdb81fe |
|  | 2a3fb28a-79e8-4abd-94f8-54f4a3b3f132 | 1658fd3a-39c9-4213-b97b-f87d04c603f8 | 36799ea3-d10a-4ec6-80bf-c851e3bbe45b | 0e03c468-afaa-4e7d-8348-774579618e1b | 23c47c82-9400-4a4f-8353-467dcee8214b | 191e1b09-6cba-48d1-aeec-e36bd5e899c9 | 7b16e5e1-cd3e-4146-bda4-104ac993375e |
|  | 2ab6d1fe-6acd-44ba-b11a-6a92612a0560 | 166203ec-93c4-45cb-9906-8fbc8156d388 | 3c7c996e-cba6-4e96-865e-559590f4ee89 | 0fe2ac2c-bd69-4d93-8f63-7d9ba73ecb36 | 24ab6eba-9099-4caa-bdd8-4660d8e549f5 | 1ad0524e-4bb2-4a64-a7b2-9f67bd09301a | 10bb810e-9ab5-4e47-9467-abbfea4462d5 |
|  | 2d7f9463-1789-46c0-a585-d8e4b3db974e | 17b53839-6c06-48cd-8829-0dd7499eec4e | 433dda04-6c51-4674-970e-1fc7c06ff99d | 100c7c39-7954-4374-bc2f-17791f5418fc | 25affd0b-eb31-4641-a779-a4f51e64e11f | 1b4915fa-7d36-477a-8bdf-30a6ce5437e9 | 11ca1c13-0187-4b52-8fea-53326094754b |
|  | 2e97bf3d-8729-4ea7-95cf-7eddfcfe2ff8 | 19943aa8-5fe4-475c-8398-529ce6c0a3c3 | 45590173-e7f9-4173-804c-8b59028b62e4 | 1029cad9-e331-4112-81f1-42f3253e282c | 26f870b5-9d30-4429-9b9f-44f663156f1b | 1c5f1e8f-e9e8-4cfe-af57-57b7618938ca | 134f9e0b-1a7a-4014-a7a7-703ae7b9fe9a |
|  | 2f6bc1f9-6b44-4066-a034-b90b9af618d8 | 1a7c071e-3191-4766-9b2f-df1d2c5f46c7 | 46500fd9-4ac4-4038-90ab-f0b4a12c6baa | 1088150d-6db6-4564-9b07-9aae6032e1a9 | 272ab114-d1bf-4197-961a-d51e66181b55 | 1c6a4b56-3e59-46e7-87bc-c988bef322ed | 13982d5a-9665-408f-b1fa-8a9eeddc1786 |
|  | 2fb9218c-5326-4de9-b847-da2917e4a803 | 1c311e03-46af-4c35-978b-cbbd8fa725e9 | 48675b8f-e65e-422e-94aa-ae00067843cb | 10a645c8-ba36-4bc8-b941-3a3b08ce231f | 2b76c244-8299-4386-869a-db13f3dca1f1 | 1df3c946-9003-4f67-aa1d-6e3746ff945b | 15b3a77a-79bc-4f07-ae94-fb38191a949a |
|  | 3199eddb-a9ea-4b44-857e-823d6070680b | 1cae5825-2dbe-4eda-a71f-40ea7a554420 | 49533bf3-4e89-42c7-a976-9b71930fb1b5 | 11055308-6551-4c31-8d60-19d8eafbca16 | 2d56ee38-c3a2-46c7-9e41-ae6eb0921e2d | 1e83d6e5-16e8-421e-a65c-055058f3fdc3 |  |
|  | 3577ddbf-fffe-4a36-980b-2c8eb02caf87 | 1cbd6458-7371-4a79-b4a9-e32894d0fa32 | 4a8afd9c-37bc-472e-b56a-3bd17c346481 | 11457950-2d57-466f-8990-92d07c47a529 | 2f646ca9-0fa3-4a01-a7d7-e61a6719a18d | 1f0a0b54-9d8a-47fa-8fe5-87a71370f7cd |  |
|  | 35dce423-671d-4621-8de6-2012636ef624 | 1d1d18b8-76d7-473e-bfc5-06d0a4f53a5d | 4ae9207f-8add-4008-86a4-f6a50e086f39 | 117edf6d-be46-49ae-9eae-83c0ee347d85 | 31d6378b-5d30-41af-869e-7343c2a24431 | 20384a2a-9149-4abd-9d1d-01d93c580f93 |  |
|  | 35f0577a-8882-4532-8603-7840671a9b6b | 1d47e720-1a02-45f4-b0dc-99861916e3e1 | 4ce411c7-5fe3-4064-b28c-ccf7650ef362 | 12648c25-a55c-4bfc-9b11-f9f2400f0fb6 | 31f3dd9e-19dd-4f1f-9246-d467e0ace6d6 | 2086a7c7-4e29-40fe-bb8d-05baf636f516 |  |
|  | 3a1d0f35-91f7-4c8a-ac51-5e4f5c0ffed1 | 1e325442-a40b-4f63-861a-ea4083cf7099 | 535bb10e-f0e5-4364-8acb-d4fac9986e5b | 12948bf5-eaa9-4660-9ee5-3d4ca9216c50 | 33b47e8e-c835-4e4d-a531-eba1652f32d7 | 213dce69-e10f-4ad2-b933-d1ef06c683b5 |  |
|  | 3c427d28-5296-40a6-8c80-07464026030e | 1e60fa48-4c11-467e-b41f-45eff7b6af08 | 54c90c19-dfae-4d63-a6e7-609b276ab615 | 12bba93b-f3df-4cba-9a66-de9fcf6f4d84 | 349b5262-bc60-47d0-8c1f-36990f455ffe | 242397d5-54af-4129-96a6-8874da96e6ed |  |
|  | 42772e6b-8a70-463e-8c8f-f2217d934deb | 2007f47e-b3aa-42b2-b7e1-9d5f0da934bf | 5594d20f-b78f-4593-b44a-65f8a749a730 | 131be75f-a780-44ac-8f1e-9c1e77f34835 | 3776ea7d-6e59-45b5-aa1b-45e1b072d0b7 | 242792d7-bf93-4db6-b338-f5c1c29177a2 |  |
|  | 4605e642-d454-4c3a-be86-7bf0c5b2d822 | 205a5aac-2040-4408-846d-19228ae435bf | 5858b342-26e7-4e14-864d-08737e1eb55a | 13c6eb81-ad86-4cac-9df8-cef9b22bc23c | 3b73adbf-d709-4971-b40c-0b0601f70dfe | 262e9414-6070-488b-8449-75d11083431e |  |
|  | 47aaf4e5-a682-4a48-b4ed-00426f424352 | 21589a6c-f540-4927-ae32-cc626844aa9c | 5945eff3-2698-4898-94a2-9868651d3ae6 | 1414f416-34a2-424e-8b54-931e17929cd8 | 3e0cc274-a0bf-4fb7-a20b-a884aa60d56d | 27021eee-7d29-4e12-9e62-94b26ba00a4a |  |
|  | 482808ff-4865-4f02-8341-33728eac3a93 | 21abffb3-3e07-4022-86ff-51a41036cfc4 | 5ee6b9ea-7828-4631-bd81-52e4ddebff4e | 1527bf2a-f5cc-486a-b258-126a58689ab3 | 43ecb481-c1de-4e55-9b15-d7e4dc5252e4 | 27c6c858-77f9-46e4-ad64-86910da79770 |  |
|  | 4930b76c-59d5-4833-88cf-072cc3777462 | 22879c27-d2a7-43cb-bebf-4fb3379003d9 | 5f06f29b-ddc7-43ae-a2d0-7df4349f1507 | 16efa13d-a7db-4406-9a3d-65523da606ea | 452306f7-acd5-45b5-9f81-0c8f8a97a700 | 27f49c20-2603-45a0-a260-d93661bf4e64 |  |
|  | 4c2c8ce5-b9ae-48b0-82e2-218141be08c3 | 24a2cd1a-ff32-4704-ae91-088c7a94de24 | 615d80bc-2f5a-43c4-a21d-8884626170ef | 171bb3f1-a6e0-4d45-b127-79ca1c84ebb5 | 46a7fa5e-928e-46eb-83e9-0194c40c0947 | 27fd0aaf-27f2-4070-8881-ccbad481f720 |  |
|  | 4cf347d7-40b7-4067-9cb8-e8f437fbc2ac | 24a6594d-c21e-45e3-9863-f6b7e304f21c | 030988e8-33db-45dc-9a6c-896b55f8c9fe | 17778863-27db-40a2-9ae3-eccdc723f5ab | 47691663-def0-4721-999b-f8ebf99349fa | 2816c887-04e1-4a63-9246-db2b55afcea2 |  |
|  | 4d71591d-ee5e-4e29-ba89-7693594744d6 | 252ee930-ff03-4cc7-8279-543bb5a955b4 | 08bcb7da-fba4-4170-af33-fff85b7a57e9 | 17834f6c-d0eb-4d7c-a546-0c301a996014 | 4c8c3be7-afac-4ab7-b80d-026943410277 | 28c1b0e8-f92c-45d3-9534-3404f19ff389 |  |
|  | 514f5902-3bf7-4994-9850-d3ed3a53a277 | 262e5aa4-6892-4d82-9cee-99be4b6232bc |  | 17a3af91-4bc8-40b3-be39-77b328e78099 | 4f17749e-03d2-451d-bc3b-7a177ad71bd5 | 290c7e33-5b4a-47f4-ae9f-607fc1854c24 |  |
|  | 53274f12-4fe6-491f-8eeb-1f67042b1a47 | 2683fb0e-6a84-4bff-a327-64cfb2dc3308 |  | 1920512b-1613-479a-8ba4-f41b5b9adb53 | 55caeb6c-9123-4f97-a373-d66148d9fb25 | 2a367699-a322-4084-8f7f-1ae813be4af2 |  |
|  | 5419fbb4-0296-4bd6-9a19-9f03fac93bbd | 27ec5c6e-6119-474a-bb61-8d1310d1551b |  | 19e40b3b-79d8-4c9a-96e5-b12da717ee62 | 56fcca8f-339f-4f81-8157-7b5d3d5b753a | 2a540412-5dd8-459b-ae3d-fc876f1dbccb |  |
|  | 54f6aa55-fa38-4766-bf70-a23a05adfeb9 | 28a15efb-f538-41fd-9517-0fb86cae338b |  | 1a390098-b270-4994-8359-5a4177af41c9 | 58983bc6-136a-48ad-ae29-936fd18a2f62 | 2a7e4f3c-d028-4111-9ce4-e44e9cc21a78 |  |
|  |  | 28bfa617-bafe-40fe-ae7b-fca66584b7cf |  | 1a7b6531-739a-4a44-b05e-841574a056fb | 050a903a-7994-4ae1-96c0-e06cf2522b6d | 2b7d56d6-70cc-48ce-a17c-1abc9d2fdff6 |  |
|  |  | 28d07a0a-b5ea-4ec0-b831-e80e060ef49c |  | 1b2e8683-2a9a-4f16-84e3-cbd267e5f5c1 | 052a2b32-c3cc-4967-b942-1f6272985939 | 2bd1f869-89bc-47af-aa77-c0ef1b2c1ce9 |  |
|  |  | 2932217e-1907-4729-82b0-f05d497fc783 |  | 1b3b5256-eaf0-4cf8-a9ae-803d473a1063 |  | 2c1cfbcb-c947-488e-891c-70559bf628a8 |  |
|  |  | 2bb3034f-d37a-4eb4-b633-a3952ab947dd |  | 1b7a497a-98d7-4e7d-822a-9d9607e732a2 |  | 2e83bcf1-5488-4b37-ada5-4e93673c1049 |  |
|  |  | 2bcb932d-2829-49a0-a30c-083ac9ff5c40 |  | 1b91c421-a3f9-4c67-95dd-5aa151e220c6 |  | 30848026-1c30-4759-9d49-d394aa03e370 |  |
|  |  | 2c658173-1941-45fe-baf5-ab67dfbcdfff |  | 1b9990e7-7a03-4fe5-ba0d-3a82c4ce0607 |  | 31b0be47-eb3c-4c74-b8df-66af6ba6e0e9 |  |
|  |  | 2d164199-c4f0-48ca-90bd-5f363b28cc58 |  | 1becbc6c-aea3-4663-a574-c6783bd2d929 |  | 3798bc64-ecde-4524-973d-9adbc9f21fd8 |  |
|  |  | 2d2554be-a947-4b7c-90b8-be1b4a0bd943 |  | 1c9745d7-0f1a-4197-a523-99238ec48ba0 |  | 39520f40-0c75-4c36-9644-d92cea1586a2 |  |
|  |  | 2da979d1-6b8f-4e13-9c51-24a1ac569935 |  | 1c9d281e-848a-423d-b988-b1716b5ded54 |  | 39b525ee-5a60-43f2-aeea-16fb79ee1be2 |  |
|  |  | 2db0a3fd-2399-4bfb-b676-2b19c197f391 |  | 1cb56a2b-3322-4e52-bb02-4fcd5fb794ad |  | 3a5d0994-5ee1-45d8-8838-6be693f7c7ee |  |
|  |  | 2e1de344-442c-4c2b-929a-74021d92c95b |  | 1dc6133f-8769-4857-add0-cbad0c232e2a |  | 3a83fd90-8a67-41d5-86e8-8e6fb8cfebbb |  |
|  |  | 301711d1-3725-4d5e-95f2-ea14ff5f9e5d |  | 1e6467e8-3420-462d-81ee-83c4691e621b |  | 3af1022c-e45f-4416-9977-ad9746dfcc47 |  |
|  |  | 30843a4c-9ac8-4f2c-85ec-f33b87e716ae |  | 1eadfa64-53dd-4e0e-b10d-b55292758ecf |  | 3afe1389-f5ea-4187-99c0-d0e5899fefb9 |  |
|  |  | 309f6763-b665-495e-91b2-07007d5cdf04 |  | 1ed7cfa5-c91b-4090-bd4a-778c74514277 |  | 3c3a8f79-e4b4-4306-b562-723c17a54138 |  |
|  |  | 31fc1006-d826-4d63-8fe0-881cce64b722 |  | 1ef14d67-4d84-4984-9336-f27e083dfe05 |  | 3d257b5d-63f0-44b0-a6a7-7d1bd99e4858 |  |
|  |  | 32bb2085-8247-4f0c-92fd-cf5bf033daa5 |  | 1f1abea9-4805-46da-8199-905c19849ebe |  | 3db134ea-4608-401a-8ef3-b2c43a6204b6 |  |
|  |  | 33f500d7-0967-4bbf-9b40-d1d6ff8e3e3a |  | 1f50dda5-20df-4a57-91ae-c4d6a1cd1f33 |  | 3db4d2ab-0453-4f7b-b2ce-726b9270c185 |  |
|  |  | 347a8e69-dab4-4920-a395-8ee80256d0ff |  | 1f6f865d-7c92-408c-a525-0439d9c086f2 |  | 3f57f1de-1c0b-4613-9c6e-4918493a5bbc |  |
|  |  | 36091e7d-21aa-4471-bd96-8fd4d93c03ff |  | 20176497-8b9c-408d-82c3-efb5ddafbb07 |  | 4016c7b2-c50f-4578-beb6-6c2d535b7d85 |  |
|  |  | 37409434-dc17-4345-96d8-5cd388d90166 |  | 2066ae7c-e6d4-4c49-a264-d55c762cd9fd |  | 40933dff-088a-489c-bbad-ff39d8c72612 |  |
|  |  | 38d4869b-6186-4af8-bac6-8fbf613b5861 |  | 20892d81-3b1a-43cc-89b2-35387f0abde5 |  | 434b15f7-52a1-4730-af14-f7359980b6ff |  |
|  |  | 3951e52f-ee5a-4a71-9ed3-a112c97cca84 |  | 20ba95d6-4bfc-4e78-b305-2ea0024b52e2 |  | 4555a3d7-4efe-4c90-ac01-46350518fff9 |  |
|  |  | 3ba60bf7-69de-49f1-b4c1-9277372c3dd7 |  | 2164812d-9725-4c7f-a153-2f0fb6477984 |  | 4649891f-7658-4628-8902-cd059029a8de |  |
|  |  | 3d03ea94-ab5e-45d9-ad13-0b0e6af4ab69 |  | 2178d10d-15e4-4e66-855a-866eac44d06f |  | 48003b32-52f9-48a3-9adf-b5e6694f91ef |  |
|  |  | 3e4b3c1f-cbb8-487f-9c9e-01291970ade3 |  | 22667b5b-e452-4917-b528-1f01ebb1d3a7 |  | 49527f1a-04a8-484d-91b8-f25b17267310 |  |
|  |  | 3e824571-a3da-4d80-98d1-c161f569466d |  | 22dc91f6-450c-4e99-9c46-8a8d1d4720bc |  | 49e510cd-72ed-43ba-b9f0-da9c795b1b6c |  |
|  |  | 3f460a1a-f1dd-4c11-95b1-f5757a8e7cc4 |  | 23606ae0-4651-4fae-8603-31627bcf44e0 |  | 4ba26854-e60e-4434-8cde-ad6a702b0e4c |  |
|  |  | 3f543d08-e293-4892-b2f2-810a937898c1 |  | 2388d8ee-26b3-4ea0-be18-6b07fa403ed0 |  | 4bb17833-16fa-4605-bea6-d846e8076c07 |  |
|  |  | 4054a92c-e4c6-4742-99fc-2e9bc0b99ed5 |  | 251c691d-2778-4d91-9357-9d6bfb2bfed6 |  | 4d17c244-a4a0-4fdc-af77-7927396c1d2a |  |
|  |  | 413308af-8010-4fa7-b7d6-b7257d73e3c8 |  | 256f97c7-bac1-440b-9153-d0cad9f6f203 |  | 510bed41-64ec-40f1-bb80-9b2e26d41f96 |  |
|  |  | 44326a5f-517c-4e99-a8ac-eea1bdf44eeb |  | 2660dc12-50d6-4629-9bed-0338a90b75d1 |  | 51dfa3d8-6b27-4c87-b76d-29a0c5c19e6c |  |
|  |  | 46113f4d-f105-4057-9ae7-27f4e0f60abe |  | 26ff1ce5-2dc7-4803-9f73-960e1d6ddc02 |  | 51e59ef0-6e17-4103-ac4f-eac9d1ad64ee |  |
|  |  | 4875a959-464d-4996-aa2b-efde14601743 |  | 277ca438-3a28-4306-926a-b283c3132804 |  | 52792628-b48a-4068-878c-960d5f6ebbcd |  |
|  |  | 4a069c77-35c2-4745-9276-333425731104 |  | 27a02f4e-84da-4d99-bf07-47e18bf6e80f |  | 54aed217-98a4-46e1-8f62-5969a8519d9d |  |
|  |  | 4aa26af5-b589-49f4-ab6f-e00fda1ea56c |  | 27ba8c96-0b30-4533-8a92-ee77cff6c27b |  | 54b6a7b4-4d2d-482e-abb3-fbbf92087cb0 |  |
|  |  | 4b08af50-9a8f-48a8-9fd5-e4d3e963dabc |  | 283801ed-ba67-4744-a916-3001c2b4fb3a |  | 54c1cf40-c66e-4a2f-b5ed-f77549f62cc9 |  |
|  |  | 4d45722d-19a0-4540-9a2b-fb4269da9caa |  | 28665a83-f3fe-4ebc-a421-2ab1b40ec796 |  | 55c2f0c9-747b-4b1a-a84f-221f84898531 |  |
|  |  | 4d4bb419-1f68-4c6e-92dc-bcfc0cc7c8e8 |  | 28e06630-d3c6-4264-9e51-62f677e9bcb6 |  | 563b1f34-57e1-4484-ac21-2a6ab29bccd1 |  |
|  |  | 4d6a370d-5a30-4f34-b8ff-d4490afcec73 |  | 28f81839-aab1-434e-8ceb-7733f70e7a0a |  | 56f851eb-ecd1-4ccd-90f8-a3574d270c51 |  |
|  |  | 4db49f5a-4675-41f2-af9b-66cd8cab43cd |  | 293299b9-f424-4bc1-9e0d-4a8700220c2b |  | 578decc2-8a64-44c2-85c3-78c6c2251149 |  |
|  |  | 4dcf0fd6-a7eb-4f62-add4-e54905acf3b9 |  | 29388e3e-0012-4ebc-8b03-0845c5d509f1 |  | 57ed6024-3192-4a71-945c-2d0c4ecf50b7 |  |
|  |  | 4e4b13ec-bfb9-4e97-9210-1974b41a3e4d |  | 29799227-de02-4309-9ee0-0365f912e0ab |  |  |  |
|  |  | 4f8f52e4-1a81-42a0-88a5-41e87b6be678 |  | 29ba7526-b0f9-469e-ad33-f01e34f3fb3c |  |  |  |
|  |  | 50db711e-b2eb-4b33-ae9f-48ce6d860a48 |  | 2a2ec9f0-ed6e-40b4-a901-c0e8817f53f5 |  |  |  |
|  |  | 5125a5a9-aeb1-4132-bd34-18d8eb38b61e |  | 2aabcff2-a8a0-4735-92c5-e0baf2f0c758 |  |  |  |
|  |  | 51c4a595-ad63-4ee2-9d5c-1ab9d398e88f |  | 2b47820b-09be-416c-8cb3-699d26a99300 |  |  |  |
|  |  | 53601c4d-ee9a-4e2a-a1ca-8d6f79f7785f |  | 2be2371f-ff0f-48ad-97e2-839b8c7a163d |  |  |  |
|  |  | 540f5a9f-330b-4817-baa6-490b83237292 |  | 2c033562-4228-4e16-85d8-8ee3d4303eab |  |  |  |
|  |  | 553abd08-4f08-4084-8d2e-8a56d6d6e120 |  | 2c77bacc-78ea-41c3-a6f6-919aa5392075 |  |  |  |
|  |  | 5608dff0-72ab-468c-b25f-d411ad581052 |  | 2d7f32a4-9add-49f4-a342-72e47db86f1f |  |  |  |
|  |  | 56a7095e-29b1-4c29-b1ed-9b5a18b36a2c |  | 2f087e96-3e8d-41c3-8cfd-9a16ca3e9c65 |  |  |  |
|  |  | 56b17f0f-88ff-4b8e-9650-026e7e118239 |  | 2fa2ab18-c0ee-4162-97b4-95dca87655d3 |  |  |  |
|  |  | 57a5ea8d-a6eb-416b-9ae3-9ceac2814e3a |  | 312036e2-004c-44bf-9bd7-96248f7618e7 |  |  |  |
|  |  | 5850111a-db51-4bf0-96ba-fda0423d654a |  | 317b07a2-0f1a-47a3-8465-184bbe2029da |  |  |  |
|  |  |  |  | 31dc61c4-f282-48bb-8bcc-986e25c587be |  |  |  |
|  |  |  |  | 3225ce49-a4a4-4554-a458-e14c2a8e0052 |  |  |  |
|  |  |  |  | 32265a11-9e95-4fa1-ba44-035993305452 |  |  |  |
|  |  |  |  | 322badf4-b70d-4f90-ac44-d2f30e52f03c |  |  |  |
|  |  |  |  | 323cc1a2-906d-4c5a-b332-a8141f6e210b |  |  |  |
|  |  |  |  | 328f0864-cde9-4bf2-9be4-b19a699575c5 |  |  |  |
|  |  |  |  | 33fc60e6-fe0d-4216-bb94-17355d7a661b |  |  |  |
|  |  |  |  | 000c6bb3-c47d-49a1-ad0f-57093521f922 |  |  |  |

**Supplementary table 2. The file ids for the tumour-matched control samples of the seven different cancer types from GDC portal** (both tumour and control from same patient sample has same file id)

| Name | Prostate  cancer | | Breast  cancer | | Head and Neck  cancer | | Kidney  cancer | | Liver  cancer | | Lung  cancer | | Uterus  cancer | |
| --- | --- | --- | --- | --- | --- | --- | --- | --- | --- | --- | --- | --- | --- | --- |
|  | Fold change | p-value | Fold  change | p-value | Fold change | p-value | Fold change | p-value | Fold change | p-value | Fold change | p-value | Fold change | p-value |
| hsa-mir-1269a | 3.004 | 0.0194 | 5.175 | 0.0382 | 3.997 | 0.01755 | 2.574 | 0.0231 | 5.855 | 0.0398 | 6.440 | 0.02615 | 7.882 | 0.03595 |
| hsa-mir-1269b | 1.747 | 0.0017 | 4.839 | 0.026 | 4.873 | 0.0471 | 2.708 | 0.01495 | 6.673 | 0.03775 | 7.877 | 0.0501 | 6.238 | 0.026 |
| hsa-mir-182-5p | 1.875 | 0.00115 | 2.264 | 0.031 | 1.043 | 0.03055 | 2.012 | 0.032 | 3.470 | 0.02555 | 2.342 | 0.0264 | 4.146 | 0.01735 |
| hsa-mir-183-5p | 1.891 | 0.00105 | 2.875 | 0.00265 | 1.418 | 0.0157 | 2.033 | 0.0175 | 3.976 | 0.04605 | 2.802 | 0.0325 | 4.972 | 0.02745 |
| hsa-mir-19a-5p | 1.048 | 0.01925 | 1.142 | 0.0404 | 1.074 | 0.04945 | 1.520 | 0.00405 | 1.601 | 0.02635 | 1.617 | 0.02805 | 1.436 | 0.02695 |
| hsa-mir-210-5p | 1.290 | 0.00095 | 3.057 | 0.0028 | 2.413 | 0.02615 | 2.683 | 0.0235 | 1.006 | 0.0273 | 4.595 | 0.0273 | 3.184 | 0.02815 |
| hsa-mir-3662 | 1.003 | 0.001 | 2.731 | 0.0331 | 1.993 | 0.00005 | 1.894 | 0.02725 | 3.037 | 0.03005 | 3.718 | 0.0257 | 2.416 | 0.029 |
| hsa-mir-147b | 1.134 | 0.00065 | 2.516 | 0.0311 | 1.019 | 0.0333 | 1.059 | 0.011 | 1.825 | 0.005 | 3.892 | 0.03505 | 2.220 | 0.03875 |
| hsa-mir-4746-5p | 1.130 | 0.00025 | 1.419 | 0.024 | 1.681 | 0.03605 | 2.336 | 0.02461 | 2.653 | 0.03935 | 1.326 | 0.0405 | 2.549 | 0.0126 |
| hsa-mir-7-5p | 1.322 | 0.00045 | 1.188 | 0.03795 | 0.618 | 0.04105 | 0.387 | 0.0431 | 0.003 | 0.0292 | 1.714 | 0.0176 | 1.128 | 0.0441 |
| hsa-mir-93-5p | 1.728 | 0.00005 | 1.097 | 0.0406 | 1.230 | 0.028 | 2.209 | 0.0175 | 1.751 | 0.0427 | 1.229 | 0.0287 | 1.601 | 0.0423 |
| hsa-mir-96-5p | 1.915 | 0.0001 | 3.226 | 0.034 | 1.339 | 0.0008 | 1.679 | 0.00134 | 3.870 | 0.0073 | 3.030 | 0.0166 | 4.337 | 0.03295 |

**Supplementary table 3. Common miRNAs and their fold change in expression across seven cancer types.**

| **Name** | **Prostate cancer** | **Breast cancer** | **Head & Neck cancer** | **Kidney cancer** | **Liver cancer** | **Lung cancer** | **Uterus cancer** |
| --- | --- | --- | --- | --- | --- | --- | --- |
| ACVR1B | 1.026 | 1.178 | 1.01 | -1.00649 | - | 1.018 | - |
| AKT3 | -1.013 | -1.23 | -1.055 | -1 | 1.235 | -1.39617 | -3.5164 |
| BCL2 | -1.23771 | -1.891 | -1.122 | -2.3679 | -1.033 | -1.44345 | -2.23857 |
| CCND1 | 1.018 | 2.621 | 1.367 | 2.027314 | - | - | - |
| CD38 | -2.12266 | -1.029 | -1.08 | -1.45149 | -1.42917 | -1.134 | - |
| CREB1 | 1.002 | 1.294 | -1.042 | 1.014 | - | - | - |
| EGFR | 1.026 | 1.084 | 1.18 | 1.237308 | 1.085 | 1.158 | 1.167412 |
| FOXO1 | -1.125 | -2.77 | -1.079 | - | -1.87601 | -1.12698 | -1.71558 |
| IGF1R | 1.009 | 1.864 | 1.022 | 1.005 | -1.059 | - | -1.01057 |
| JUN | -1.016 | -2.167 | -1.011 | -1.027 | -1.61383 | -1.10336 | -1.51554 |
| KRAS | -1.002 | 1.45 | 1.072 | 1.046 | - | - | - |
| MAP2K2 | -1.004 | 1.469 | -1.051 | 1.02 | - | - | - |
| MAP3K3 | 1.013 | -1.111 | 1.031 | 1.014 | 1.076 | -1.09193 | -1.48488 |
| MAPK1 | -1.004 | 1.509 | 1.023 | 1.013 | -1.012 | - | - |
| MAPK8 | -1.015 | -1.072 | -1.022 | -1.08734 | - | -1.034 | - |
| MAPK9 | 1.017 | 1.374 | -1.084 | 1.17 | 1.06 | - | - |
| MYC | 1.079 | -1.113 | 1.217 | 1.721876 | -1.53212 | 1.209 | -1.39724 |
| MYLK3 | -1.005 | -1.108 | 1.005 | 1.036 | - | - | - |
| NRAS | -1.014 | 1.467 | -1.051 | -1.023 | -1.017 | - | - |
| PIK3CG | 1.042 | 1.401 | 1.051 | 1.113632 | - | -1.01951 | -1.04976 |
| PRKACB | -1.025 | 1.057 | -1.029 | -1.028 | -1.021 | -1.017 | -1.32413 |
| RAF1 | 1.015 | 1.04 | -1.174 | -1.286 | - | - | - |
| RELA | 1.022 | 1.236 | 1.057 | 1.012 | - | - | - |
| SCD | -1.009 | -1.584 | -1.034 | 3.370612 | -1.074 | -1.21325 | 2.836245 |
| SMAD4 | -1.067 | -1.256 | -1.116 | - | -1.044 | -1.104 | - |
| SNAI2 | -1.82177 | -1.896 | 1.152 | - | - | - | -2.48995 |
| VEGFA | 1.006 | 1.844 | 1.007 | 3.276695 | - | 1.061 | - |
| XIAP | - | 2.349 | - | 1.077 | - | - | - |

**Supplementary table 4. The key genes as targets for the common miRNAs along with their fold change in expression in the seven cancer types.**

| ***Name of the miRNAs*** | ***Type*** | ***Reference*** |
| --- | --- | --- |
| hsa-mir-182-5p | Exosome | 1–3 |
| hsa-mir-183-5p | Exosome | 1–4 |
| hsa-mir-210 | Circulating and exosome | 1–3,5–8 |
| hsa-mir-19a | Circulating and exosome | 1,4,9,10 |
| hsa-mir-7-5p | Circulating and exosome | 2,6,11 |
| hsa-mir-3662 | Circulating | 12 |
| hsa-mir-93 | Circulating and exosome | 6,11,13–15 |
| hsa-mir-519d | Circulating | 8,16–18 |
| hsa-mir-524 | Circulating | 8,15 |
| hsa-mir-548b-5p | Circulating | 19,20 |
| hsa-mir-208a | Circulating | 15,21–24 |

**Supplementary table 5. List of circulating and exosomal miRNAs as obtained from Mirandola database.**


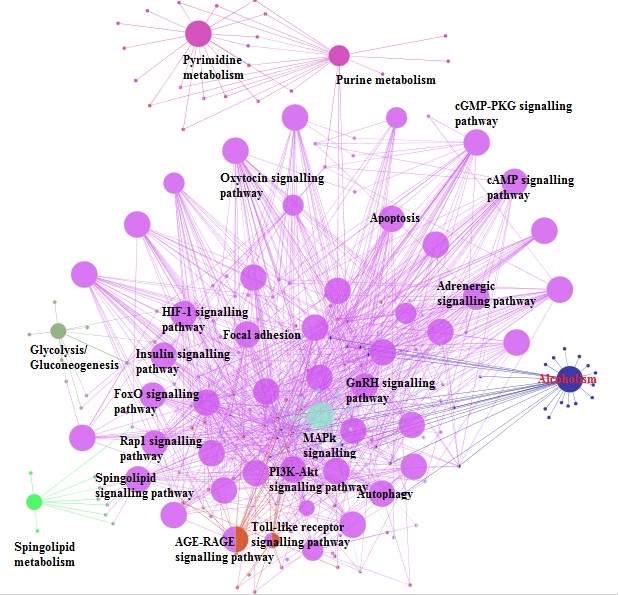


**Supplementary figure 1**: **Network analysis: Validation of pathways regulated by common miRNAs using ClueGo.** The ClueGo analysis of the target genes regulated by the common miRNAs. Colours (Green, Olive, Pink, Purple, Cyan and Blue) indicate different network groups formed by the target genes in a pathway. The groups with purple and red colour indicate post-transcriptional modification and inhibitory regulation within the genes of the group.

References

1. Taylor, D. D. & Gercel-Taylor, C. MicroRNA signatures of tumor-derived exosomes as diagnostic biomarkers of ovarian cancer. *Gynecol. Oncol.* **110,** 13–21 (2008).

2. Mittelbrunn, M. *et al.* Unidirectional transfer of microRNA-loaded exosomes from T cells to antigen-presenting cells. *Nat. Commun.* **2,** (2011).

3. Cheng, L., Sun, X., Scicluna, B. J., Coleman, B. M. & Hill, A. F. Characterization and deep sequencing analysis of exosomal and non-exosomal miRNA in human urine. *Kidney Int.* (2014). doi:10.1038/ki.2013.502

4. Hunter, M. P. *et al.* Detection of microRNA expression in human peripheral blood microvesicles. *PLoS One* **3,** (2008).

5. Rabinowits, G., Gerçel-Taylor, C., Day, J. M., Taylor, D. D. & Kloecker, G. H. Exosomal microRNA: A diagnostic marker for lung cancer. *Clin. Lung Cancer* **10,** 42–46 (2009).

6. Mitchell, P. S. *et al.* Circulating microRNAs as stable blood-based markers for cancer detection. *Proc. Natl. Acad. Sci.* (2008). doi:10.1073/pnas.0804549105

7. Ho, A. S. *et al.* Circulating miR-210 as a novel hypoxia marker in pancreatic cancer. *Transl. Oncol.* **3,** 109–113 (2010).

8. Gui, J. *et al.* Serum microRNA characterization identifies miR-885-5p as a potential marker for detecting liver pathologies. *Clin. Sci.* **120,** 183–193 (2011).

9. Moltzahn, F. *et al.* Microfluidic-based multiplex qRT-PCR identifies diagnostic and prognostic microRNA signatures in the sera of prostate cancer patients. *Cancer Res.* **71,** 550–560 (2011).

10. Wang, Q. *et al.* Plasma miR-601 and miR-760 Are Novel Biomarkers for the Early Detection of Colorectal Cancer. *PLoS One* **7,** (2012).

11. Wulfken, L. M. *et al.* MicroRNAs in renal cell carcinoma: Diagnostic implications of serum miR-1233 levels. *PLoS One* **6,** (2011).

12. Wang, H., Peng, R., Wang, J., Qin, Z. & Xue, L. Circulating microRNAs as potential cancer biomarkers: The advantage and disadvantage. *Clinical Epigenetics* **10,** (2018).

13. Moussay, E. *et al.* MicroRNA as biomarkers and regulators in B-cell chronic lymphocytic leukemia. *Proc. Natl. Acad. Sci. U. S. A.* **108,** 6573–6578 (2011).

14. Zahm, A. M. *et al.* Circulating microRNA is a biomarker of pediatric crohn disease. *J. Pediatr. Gastroenterol. Nutr.* **53,** 26–33 (2011).

15. Goren, Y. *et al.* Serum levels of microRNAs in patients with heart failure. *Eur. J. Heart Fail.* **14,** 147–154 (2012).

16. Weber, J. A. *et al.* The microRNA spectrum in 12 body fluids. *Clin. Chem.* **56,** 1733–1741 (2010).

17. Yang, Q. *et al.* Application of next-generation sequencing technology to profile the circulating microRNAs in the serum of preeclampsia versus normal pregnant women. *Clin. Chim. Acta* **412,** 2167–2173 (2011).

18. Zhao, Z. *et al.* Circulating microRNA miR-323-3p as a biomarker of ectopic pregnancy. *Clin. Chem.* **58,** 896–905 (2012).

19. Xiao, J. *et al.* MicroRNA-134 as a potential plasma biomarker for the diagnosis of acute pulmonary embolism. *J. Transl. Med.* **9,** (2011).

20. Yang, C. *et al.* Identification of seven serum microRNAs from a genome-wide serum microRNA expression profile as potential noninvasive biomarkers for malignant astrocytomas. *Int. J. Cancer* **132,** 116–127 (2013).

21. Wang, G. K. *et al.* Circulating microRNA: A novel potential biomarker for early diagnosis of acute myocardial infarction in humans. *Eur. Heart J.* **31,** 659–666 (2010).

22. Fichtlscherer, S. *et al.* Circulating microRNAs in patients with coronary artery disease. *Circ. Res.* **107,** 677–684 (2010).

23. De Rosa, S. *et al.* Transcoronary concentration gradients of circulating MicroRNAs. *Circulation* **124,** 1936–1944 (2011).

24. Xiao, J. *et al.* Serum microRNA-499 and microRNA-208a as biomarkers of acute myocardial infarction. *Int. J. Clin. Exp. Med.* **7,** 136–141 (2014).
